# Telemedicine for diabetes management during COVID-19: what we have learnt, what and how to implement

**DOI:** 10.3389/fendo.2023.1129793

**Published:** 2023-05-17

**Authors:** Laszlo Rosta, Adrienn Menyhart, Wael Al Mahmeed, Khalid Al-Rasadi, Kamila Al-Alawi, Maciej Banach, Yajnavalka Banerjee, Antonio Ceriello, Mustafa Cesur, Francesco Cosentino, Alberto Firenze, Massimo Galia, Su-Yen Goh, Andrej Janez, Sanjay Kalra, Nitin Kapoor, Nader Lessan, Paulo Lotufo, Nikolaos Papanas, Ali A. Rizvi, Amirhossein Sahebkar, Raul D. Santos, Anca Pantea Stoian, Peter P. Toth, Vijay Viswanathan, Peter Kempler, Manfredi Rizzo

**Affiliations:** ^1^ Primary Healthcare Centre, Felsorajk, Hungary; ^2^ Department of Medicine and Oncology, Semmelweis University, Budapest, Hungary; ^3^ Heart and Vascular Institute, Cleveland Clinic, Abu Dhabi, United Arab Emirates; ^4^ Medical Research Center, Sultan Qaboos University, Muscat, Oman; ^5^ Department of Training and Studies, Royal Hospital, Ministry of Health, Muscat, Oman; ^6^ Department of Preventive Cardiology and Lipidology , Medical University of Lodz (MUL), Lodz, Poland; ^7^ Department of Medicine, Polish Mother’s Memorial Hospital Research Institute (PMMHRI), Lodz, Poland; ^8^ Cardiovascular Research Centre, University of Zielona Gora, Zielona Gora, Poland; ^9^ Department of Biochemistry, Mohammed Bin Rashid University, Dubai, United Arab Emirates; ^10^ Istituto di Ricovero e Cura a Carattere Scientifico (IRCCS) MultiMedica, Milan, Italy; ^11^ Clinic of Endocrinology, Ankara Güven Hospital, Ankara, Türkiye; ^12^ Unit of Cardiology, Karolinska Institute and Karolinska University Hospital, University of Stockholm, Stockholm, Sweden; ^13^ Unit of Research and International Cooperation, University Hospital of Palermo, Palermo, Italy; ^14^ Department of Biomedicine, Neurosciences and Advanced Diagnostics (Bind), University of Palermo, Palermo, Italy; ^15^ Department of Endocrinology, Singapore General Hospital, Singapore, Singapore; ^16^ Department of Endocrinology, Diabetes and Metabolic Diseases, University Medical Center Ljubljana, Ljubljana, Slovenia; ^17^ Department of Endocrinology, Bharti Hospital, Karnal, India; ^18^ Department of Endocrinology, Diabetes and Metabolism, Christian Medical College, Vellore, India; ^19^ Baker Heart and Diabetes Institute, Melbourne, VIC, Australia; ^20^ The Research Institute, Imperial College London Diabetes Centre, Abu Dhabi, United Arab Emirates; ^21^ Center for Clinical and Epidemiological Research, University Hospital, University of São Paulo, Sao Paulo, Brazil; ^22^ Diabetes Center, Second Department of Internal Medicine, Democritus University of Thrace, University Hospital of Alexandroupolis, Alexandroupolis, Greece; ^23^ Department of Medicine, University of Central Florida College of Medicine, Orlando, FL, United States; ^24^ Applied Biomedical Research Center, Mashhad University of Medical Sciences, Mashhad, Iran; ^25^ Biotechnology Research Center, Pharmaceutical Technology Institute, Mashhad University of Medical Sciences, Mashhad, Iran; ^26^ Department of Biotechnology, School of Pharmacy, Mashhad University of Medical Sciences, Mashhad, Iran; ^27^ Heart Institute (InCor), University of Sao Paulo Medical School Hospital, Sao Paulo, Brazil; ^28^ Hospital Israelita Albert Einstein, Sao Paulo, Brazil; ^29^ Faculty of Medicine, Diabetes, Nutrition and Metabolic Diseases, Carol Davila University, Bucharest, Romania; ^30^ Cicarrone Center for the Prevention of Cardiovascular Disease, Johns Hopkins University School of Medicine, Baltimore, MD, United States; ^31^ Department of Medicine, Diabetes Research Centre, Chennai, India; ^32^ Department of Health Promotion, Mother and Child Care, Internal Medicine and Medical Specialties (Promise), School of Medicine, University of Palermo, Palermo, Italy

**Keywords:** telemedicine, type-2 diabetes (T2DM), COVID - 19, glucose monitoring, diabetes prevention and control, diabetes care

## Abstract

The past two decades have witnessed telemedicine becoming a crucial part of health care as a method to facilitate doctor-patient interaction. Due to technological developments and the incremental acquisition of experience in its use, telemedicine’s advantages and cost-effectiveness has led to it being recognised as specifically relevant to diabetology. However, the pandemic created new challenges for healthcare systems and the rate of development of digital services started to grow exponentially. It was soon discovered that COVID-19-infected patients with diabetes had an increased risk of both mortality and debilitating sequelae. In addition, it was observed that this higher risk could be attenuated primarily by maintaining optimal control of the patient’s glucose metabolism. As opportunities for actual physical doctor-patient visits became restricted, telemedicine provided the most convenient opportunity to communicate with patients and maintain delivery of care. The wide range of experiences of health care provision during the pandemic has led to the development of several excellent strategies regarding the applicability of telemedicine across the whole spectrum of diabetes care. The continuation of these strategies is likely to benefit clinical practice even after the pandemic crisis is over.

## 1 Introduction

Shortly after the beginning of the coronavirus disease of 2019 (COVID-19) pandemic, it became apparent that the presence of diabetes increased the risk of morbidity (1, 2) in the setting of a COVID-19 infection. According to a meta-analysis by Mantovani et al. (3), the chances of requiring intensive care more than doubled in patients undergoing treatment for diabetes who were hospitalised with COVID-19 (n=22 studies; odds ratio: 2.10), while their risk of mortality almost tripled (n=15 studies, odds ratio: 2.6). Analysing the English diabetes registry, Holman et al. (4) reported that glycaemic control – estimated on the basis of HbA1c measurements – and BMI were independent predictors of mortality related to COVID-19 infection. They also found that, compared to patients with HbA1c levels between 6.5-7%, type 1 diabetic (T1DM) patients with HbA1c levels over 10% had a 113% increase in COVID 19 related mortality; in those with type 2 diabetes mellitus (T2DM), mortality rose by 61%. It was also shown that patients under 70 years of age were at an even greater risk. An analysis of self-monitored blood glucose (SMBG) data of Hungarian diabetic patients with concurrent COVID-19 infection revealed that there was a statistically significant correlation between increasing average blood sugar levels and the complexity of the treatment (5). At the same time, it should be noted that in cases where insulin therapy was administered 4 or more times daily, blood sugar levels remained below 10 mmol/l. This should be highlighted as the mortality rate of diabetic patients suffering from COVID-19 infection with blood sugar levels over 10 mmol/l is several times higher compared to those whose level is below 10 mmol/l (6). In addition, the publication draws attention to the importance of glucose self-monitoring, and controlling body weight in order to reduce risk of cardiovascular events and hypoglycaemia. The emphasis on social distancing and avoidance of close personal contact prioritized during the COVID-19 pandemic has created challenges in maintaining the delivery of medications and medical supplies for patients with diabetes. At the same time, the need for optimal glycaemic control and the prevention of complications became even more pronounced. As a result, the role of telemedicine, telemonitoring and teleconsultation has become more prominent.

## 2 What is Telemedicine?

The World Health Organization (WHO) defines telemedicine as a medical service provided where distance is a critical factor, and medical service providers apply different communication technologies to overcome these distance-related issues (7). The goal is to share information necessary for diagnosis, therapy, and prevention of disease. Telemedicine can also be adapted for use in research and in the education of health care professionals.

In practice, Telemedicine can take the following forms:

- Tele-consultation/supervision: the physician can remotely involve other doctors and specialists in the diagnostic and clinical management process.
- Tele-manipulation: the use of remote control/video devices, robots and sensors in examination or intervention (e.g.: endoscopy).
- Tele-diagnostics: when the person carrying out the examination and the person making the diagnosis (responsible for the medical record) are in separate locations but have an interactive connection.
- Tele-monitoring: patients are given interactive devices and sensors for remote monitoring.

Telemedicine is in fact similar to the in-person doctor-patient contact in several ways and can positively influence the patients’ sense of security, health consciousness and adherence (8, 9). It also has the potential to promote time efficiency and increases cost effectiveness (10).

In the following section we summarize the possibilities of using telemedicine in different aspects of diabetes care by introducing relevant examples.

## 3 Telemedicine and diabetes prevention

It is widely known that two main pathomechanisms play a role in the development of prediabetes: insulin resistance and beta-cell dysfunction. This means that if we can preserve beta-cell function and/or moderate insulin resistance, we can potentially delay or prevent prediabetes progression (11).

Two studies are considered milestones in diabetes prevention. The Finnish Diabetes Prevention Study (12, 13) confirmed the role of intensive lifestyle therapy, while the Diabetes Prevention Program (14, 15) was a randomized clinical trial (RCT) carried out in the USA that confirmed the preventive role of metformin alongside lifestyle therapy in the prevention of diabetes. By developing digital techniques, researchers also tried to adopt these results to telemedicine (16, 17), and these efforts have collectively become known as the digital Diabetes Prevention Program or d-DPP.

It is difficult to draw meaningful conclusions from a comparative analysis of the results of telemedicine studies because of the different media technologies utilized (phone/video consultation, SMS, email applications etc.). Nevertheless, according to one meta-analysis, patients experienced an average 4% body weight loss during a digital prevention program (18). As a result, researchers planned and initiated the Preventing Diabetes Through Digital Coaching for Translation and Scalability (PREDICTS) Trial in 2022 (19, 20). The aim of the study was to test a widely available and clinically adaptable d-DPP technique that is effective in the reduction of HbA1c, body weight and is also capable of decreasing cardiovascular risk in subjects with prediabetes. Participants in the trial were divided into two groups. The SGE (Small Group Education) group were assigned as the control group and were further split into smaller groups each receiving diabetes prevention education with a primary focus on body weight reduction.

In the d-DPP group, participants were supported by the following:

- interactive, internet-based lifestyle change promoting lectures
- virtual self-help groups
- body weight and physical activity monitoring by remote devices with continuous feedback and assessment.

After one year, participants in the d-DPP group showed significant decreases in HbA1c levels (-0,23%) (primary endpoint), body weight (-5,5%) and lipid parameters (HDL, HDL/total cholesterol) compared to those in the SGE groups. These results illustrate that with the help of d-DPP the risk factors associated with T2DM can be successfully mitigated. An obvious benefit is that telemedicine-based prevention can be effectively utilised and applied in the midst of epidemics such as COVID-19.

## 4 Telemedicine and education

For patients with diabetes, it is vitally important to be educated on both how to keep it under control and what associated health risks they may have. Healthcare systems are obligated to prepare and support patients in managing their illness and coping with related issues. This appears in the scientific literature as “diabetes self-management education and support (DSMES).” According to 2022 Standards of Medical Care from the American Diabetes Association (ADA), “the purpose of DSMES is to give people with diabetes the knowledge, skills, and confidence to accept responsibility for their self-management. This includes collaborating with their healthcare team, making informed decisions, solving problems, developing personal goals and action plans, and coping with emotions and life stresses.” (21). The fact that this process could be achieved by using telemedicine was demonstrated in 2003 (22), where education based on personal contact was compared to telemedical education (video consultation in this case). It was found that both methods were equally effective in the improvement of glycaemic control. The questionnaire survey also showed that both methods had a favourable effect on psychosocial factors. It is interesting to note that, during treatment, no problems were encountered in either group with the introduction of insulin therapy. Further meta-analyses (23, 24) also established that the application of telemedicine in type-2 diabetic patients improved both glycaemic control and DSMES.

Obesity, an important risk factor and comorbidity in T2DM, can also be treated successfully with the help of telemedicine. In the POWeR study (25), WEB based intervention was compared to personal education, and it was found that in the WEB based cases there was a 1,5 kg increase in weight loss compared to the control group. Furthermore, 30% of patients on the POWeR group maintained at least a 5% body weight loss for an additional year compared to the starting point, without further education or any additional healthcare costs.

Further investigations (26, 27) established that the most important factors determining the efficiency of DSMES based on the web include:

• encouraging self-management
• sharing personalized information
• exploring incidental difficulties and solving emerging problems
• feedback on achievement
• interaction based on the internet between healthcare providers

Based on the findings mentioned above, Poduval et al. (28) started an 8-week-long online course specifically for patients with a recent diagnosis of diabetes. In addition to basic education on diabetes, the course also focused on lifestyle change, weight loss, defining individual target values and the prevention of complications. The program was tested in several phases and was altered and adjusted based on patient feedback. Not surprisingly, participants reported the length of the program as their main challenge.

## 5 Telemedicine and diabetes care

To prevent long-term complications in diabetes, a factor of key importance in patient care is optimal metabolism control. A meta-analysis (23) reviewing the results of 32 publications concluded that in primary diabetic care, telemedicine could be a functioning alternative to, and even be used alongside, traditional communication methods. It was also reported that with the use of telemedicine, glycaemic control and patients’ self-managing abilities had improved. Intervention based on telemonitoring and mobile applications proved to be especially successful. It has to be noted, however, that the studies above have certain limitations. First, there is a high level of heterogeneity in some outcomes, which may have been due to differences in telemedicine interventions, subjects, interventionists, and the timing and frequency of the interventions. Second, there is the small number of studies reporting diabetes self-management ability, the findings are inconclusive regarding the effectiveness of self-management and they generally require large sample testing and further investigation. Third, there are few studies with long-term follow-up and the findings may contain only short-term effects of improving T2DM and they lack evaluation of long-term effects.

An excellent example of the application of the utilization of telemedicine in clinical practice is reflected in the Onduo virtual patient care help programme (29–31). The participants of this study received real-time continuous glucose monitoring (RT-CGM) devices. Using a phone application and the data gathered from the devices, a care team was able to provide education and advice on lifestyle modification tailored to the patients’ needs (32), while making necessary therapy adjustments according to the actual recommendations of the ADA (33). Type 2 diabetic patients with HbA1c levels ranging from 8-12% were selected for the purposes of the study. During a four-month period, there was a therapy modification in 87% of the patients, which resulted in an average 1.6% decrease in HbA1c levels (SD: 1.0; P: <0.001). With respect to active agents, use of DPP-4-inhibitors and sulfonylureas typically decreased, while GLP-1 receptor agonists were prescribed more frequently. In some cases, the introduction of insulin therapy was also necessary. Other telemedical programs applied in T2DM – mostly based on RT-CGM – were confirmed to be effective in reducing HbA1c even beyond the Onduo programme (34–36). However, clinical inertia is one of the main barriers to achieving optimal glycaemic control. Protracted poor metabolic control evidently enhances the risk of developing adverse complications (37). A study (38) showed that nearly 50% of patients with HbA1c levels between 8-8,9% taking two different antidiabetic drugs would not receive any therapy modifications until six months after the recognition of their unhealthy metabolic status.

Patients’ blood glucose data shared in real time with the healthcare providers as well as adequate communication between healthcare providers and patients are key factors in introducing adjustments to therapy at the appropriate time. This issue became even more important during the COVID pandemic when opportunities for surgery and personal doctor-patient meetings became remarkably limited. In this respect the lack of opportunity to control metabolic status resulted in the occurrence of adverse reactions and a higher mortality rate due to infection with SARS CoV virus.

## 6 Application of new digital technologies in healthcare

The COVID-19 pandemic accelerated the medical implementation of digital technologies. Among these belongs the so-called blockchain. Blockchain technology was introduced through Bitcoin in 2008 (39). A blockchain can be thought of as a shared (or distributed) database that is spread across multiple sites and participants. In order for new data to be added to a blockchain, they are first compiled into a “block”, which is simply a collection of records to be added to the database. The block is then combined with some data (a “hash key”) from the previous block through a cryptographic technique called “hashing” before it is added. Because it combines the previous block’s hash key, each new block is tied to all its predecessors in the form of a chain – hence the term “blockchain” (40). In health care, blockchain could serve as a replacement to traditional distributed database management systems (41) which have generally been client-server databases with Structured Query Language or relational input. Although traditional distributed database management systems are an established platform in health-care systems, they have substantial limitations such as the inability to support peer-to-peer data sharing, susceptibility to external adversaries (e.g.: hacking) and the absence of immutable (i.e., unchangeable) audit trail. By incorporating blockchain technology into healthcare services, a decentralised health care data management system could be created that coordinates on-chain events. A recent study of 2021 analysing the data of 415 publications (42) highlighted the following points:

- Blockchain technology is an effective digital technology to manage electronic medical records, with the ability to assign controlling rights to patients (43, 44).
- Application of blockchain has been explored for the management of clinical trials to potentially improve transparency and auditability (45).
- Blockchain technology could facilitate medical research collaboration, especially in the field of AI development with privacy-preserving technologies (46, 47).
- Another area of blockchain research interest is the field of the so-called omics technologies (e.g.: genomics, proteomics, and metabolomics) and genetics. This interest in blockchain technology stems from concerns about the ability of centralised databases to manage such highly confidential data (48–50).

This is relevant for patients with diabetes, a group of chronic patients prone to generating a variety of health-related data (51).

## 7 Telemedicine and diabetes care: an international perspective

In the second half of this paper, a brief overview of the implementation and utilisation of various telemedicine solutions – with special emphasis on diabetes care – will be presented in four countries: Hungary, the US, Turkey and Poland. The insights and experiences gained during the implementation of various forms of telemedicine both pre- and post-pandemic can to a great extent contribute to our better understanding of how telemedicine can be utilized effectively to best serve the needs of both patients and health care professionals in clinical practice.

### 7.1 Using telemedicine in diabetes care in Hungary

In Hungary, the use of telemedicine has been introduced in several areas of health care prior to the COVID-19 pandemic. Of these, the ECG-based remote diagnostic system introduced by the Hungarian National Ambulance Service (OMSZ) (52, 53) was the most widespread. The basis of this is a trans-telephonic ECG (TTECG), which forwards any ECG carried out by the primary care provider or emergency unit from the emergency to a 24-hour cardiac centre. After immediate evaluation, the patient is directed to the appropriate provider. In diabetology, for approximately 10 years access has been granted to digital blood glucose logs. Currently, all National Insurance supported blood glucose measuring devices have digital applications. In addition, mobile applications assisting in diabetes care are increasingly and more broadly supplied to patients.

**Figure 1:**
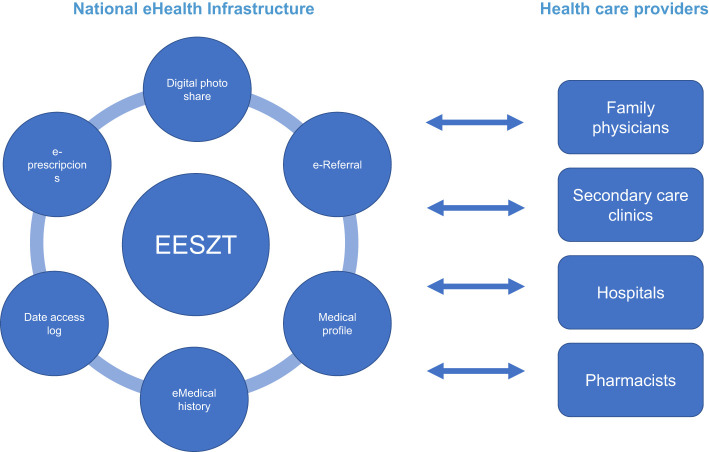
How does the EESZT (National eHealth Infrastructure) services support healthcare.

The National eHealth Infrastructure (EESZT) began functioning in November 2017 (**Figure 1**). On 1st November 2017, all primary care providers, pharmacies and publicly financed out- and inpatient institutions were obliged to connect to this new e-medical system. The OMSZ joined the system on 1st November 2018, and on 1st January 2020, adopting the system became mandatory for all licensed private healthcare providers. Although doctors and pharmacists continued to use the earlier, more familiar medical systems, IT providers were required to make the different software compatible for communication and data exchange with the EESZT. EESZT provides the opportunity for electronic prescriptions (e-prescriptions), checking the prescription fill rate and for sharing patients’ medical documents (EHR) between various medical institutions. Patients also have access to their records after entering a unique personal ID code on the Citizen Portal Site. They can request notifications if any new personal data are available, can trace who and when had access to the EESZT and if necessary, can define restrictions and permissions to access some data under the so-called digital autonomy protocols. When the epidemic began, the system was fully integrated into the healthcare system and in practice, all healthcare providers were connected to and used the EESZT. Public interest in telemedicine showed a subsequent exponential growth during lockdown and the system itself expanded and adapted to current needs. Not only could people access their medical findings and prescriptions, but they could also arrange appointments for vaccination and download copies of their vaccination certificate and COVID test results. As a response to this situation, on 30 September 2021, the Digital Health Summit Conference was held, where it was revealed that 43% of the adult population in Hungary were in fact active EESZT users and only 24% were unaware of its existence.

The Hungarian Diabetes Association (MDT) has been a committed supporter of Telemedicine for years. This is well demonstrated by the Society having a separate work group which is entirely devoted to telemedicine. MDT was able to support the work of diabetologists and assist in diabetes care in many ways during the pandemic (54). The society was in continuous discourse with the government and was able to advise on the safe continuation of medical care. Many of its suggestions have been implemented. Since April 2020, MDT and its *Primary care work group* have provided the opportunity, available *via* the society’s homepage, of online consultation for colleagues working in primary care. Experience gained during the initiative will help facilitate the full implementation of an online visit/consultation service which is to be financed by National Insurance. On 29 April 2020, the government published an edict officially regulating telemedical services (55) and in the case of eligible conditions, it is currently the recommended form of healthcare.

To promote the future provision of safe healthcare, the continuous education of diabetologist colleagues and the improvement of technical and instrumental conditions in secondary diabetology care as quick as possible, the MDT distributed a total of 260 tablets *via* a competition. These came with several preinstalled applications that assist in daily healthcare, diabetes care and communication.

The web system of the MDT has been functioning for more than 18 years and has become the number one information source and professional portal in Hungarian diabetology. Using the site became especially prominent during the pandemic as it played a vital role in both information transfer and in the provision of up-to-date guidance. It also proved vitally important as a means of organising and handling online conferences in addition to its already established role as a means of continuous education. With the help of its in-house developed mobile application, MDT has made news and professional information accessible at any time from any location. It also has the capability of sending notifications and reminders directly to mobile phones.

Another major milestone occurred in May 2021 when the website TELEMEDICINE went online. This site provides practical information and assistance to specialists, patients and for any member of the public. Its content covers a wide range of topics which are regularly updated. One can find articles and news on telemedicine, video presentations from both specialists and patients, practical guidance, and questionnaires to test one’s knowledge. There are also smart phone applications specifically targeted at the prevention, care and treatment of diabetes and opportunities for online consultation. Moreover, there is a collection of documents and articles containing useful information that patients can access.

As for the future, MDT will continue to cooperate with the relevant government departments and ministries and play an important role in improving the diabetology profile and content of the EESZT. It is currently working on the creation of a National Diabetes Register and the integration of both diabetes care protocols and digital blood sugar logs into the EESZT.

### 7.2 The evolution of telemedicine in the U.S. during the COVID-19 pandemic

In the U.S., Telemedicine assisted delivery of healthcare before the COVID-19 pandemic had been explored largely to overcome barriers of access and patient travel in remote and rural areas (56, 57). For example, in an NIH-funded trial, the delivery of group lifestyle education and retinal exams in patients with diabetes living in the small towns of South Carolina was shown to be effective (58). However, because of the large size of the country and the diversity of the population with several health care challenges, advances in the effectiveness and reimbursement of telehealth services had been spotty and fragmented. The onslaught of the COVID-19 pandemic changed both the need and the means of delivery of health care in an accelerated manner (59). Many traditional restrictions on medical care provisions and insurance coverage were summarily modified and lifted to facilitate vital care for acute as well as chronic illnesses (60). Overall, it can be safely stated that diabetes care benefitted from the collective realization that the pandemic was a highly unusual, once in a lifetime challenge of potentially catastrophic proportions that required urgent approaches, akin to a large-scale medical emergency. Diabetes education, remote glucose monitoring *via* sensors, software system utilization, and actual virtual visits with electronic portal communication were put on “fast-track” progression and coverage. Further, it was noted that there was a highly complex, bidirectional relationship between COVID-19 and diabetes; thus, when concomitantly present, the burden of both was enhanced in a vicious cycle (61). The observation of worsening of pre-existing diabetes with acute COVID-19 and the unravelling of its chronic impact in “long COVID” is clearly of major concern to the health care community (62). The debate on whether the SARS CoV virus is diabetogenic in nature and can lead to *de novo*, new-onset diabetes still rages on (63). The current landscape in this vast nation of racial and geographic differences involves diabetes care *via* telemedicine that encompasses a variety of strategies involving a combination of virtual visits, data sharing between health care professionals and patients, and electronic communication (64, 65). Fortunately, reimbursement and medical coverage rates by government-based and third-party payers remain high, and diabetes management by telemedicine-based modes appears to be here to stay in the pandemic, long COVID, and post-COVID eras (the “new normal”) (66, 67).

### 7.3 Using telemedicine in diabetes care in Turkey

The use of telemedicine in some areas of health services in Turkey began before the Covid-19 pandemic but it increased mostly after the pandemic. Diabetes mellitus is the leading area for telemedicine in Turkey. “My Mobile Diabetes” is a cloud based mobile diabetes control application which was produced and used before pandemic; it is a system that facilitates the monitoring of daily activities related to diabetes from the smart phones of patients (68). Web-based diabetes education programs were also conducted in Turkey before the pandemic. After a web-based diabetes education program was developed for patients with type 1 diabetes, there was no significant change in the HbA1c levels of the patients, while the self-efficacy of the individuals with diabetes improved and their quality of life increased (69). In another study with type 2 diabetics comparing web-based diabetes education, better metabolic results were obtained in patients using the web-based system compared to those not using it (70).

Turkish Ministry of Health supports the telemedicine in diabetes management and The Ministry has been involved in ProEmpower, a digital platform for self-monitoring individuals with diabetes (71). “Delivery of Remote Health Services” regulation, defining the compatibility of tele-health services, was published on February 10, 2022 by the Turkish Ministry of Health (72). This regulation enables healthcare providers to give services independent from geography by using written and visual communication channels. The health data of individuals could be measured and monitored with wearable technologies and medical devices.

**Figure 2:**
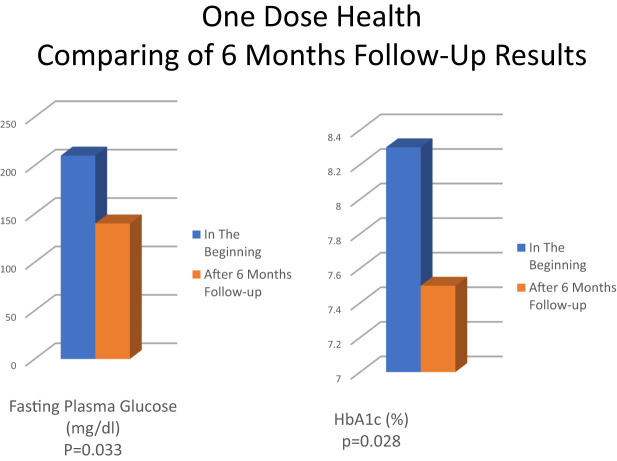
One Dose Health comparing 6 months follow up results.

During the pandemic the use of telemedicine increased in hospitals. Diabetes Digital Health Assistant is one of these services that provides remote patient monitoring with smart technologies. Blood glucose measurement is made with continuous glucose measurement sensors and falls on the screen of specialist doctors and diabetes education nurses with the software program applied (73). Another software program in Turkey is One Dose Health which is an online diabetes follow-up program developed by the Guven Future. In a pilot study of One Dose Health follow-up program conducted among diabetic patients on insulin therapy (5 Type 1, 1 Type 2), it was observed that fasting blood glucose and HbA1c values ​​were significantly better after 6 months of follow-up (74). The mean of initial and final fasting plasma glucose changed from 211.3 ± 82.6 mg/dl to 136.8 ± 41.0 mg/dl (p= 0.033), and the mean of HbA1c decreased from 8.3 ± 1.1% to 7.5 ± 1.1% (p= 0.024) and the results were statistically significant (**Figure 2**). This pilot study shows that telemedicine may allow close patient follow-up and effective patient-nurse-doctor communication in individuals with diabetes and can make a significant positive contribution to diabetes management.

### 7.4 Utilizing telemedicine in Poland in diabetes care and beyond

In Poland the widespread use of telemedicine in clinical practice and diabetes care was demonstrated by various innovative initiations in recent years. Within the framework of REPROGRAM Consortium (COVID-19 Pandemic Health System REsilience PROGRAM), Bhaskar et al. (75) argue that in order to create state-of-the-art and resilient health care systems, telemedicine and IT solutions such as artificial intelligence (AI) or robotics developed during the COVID-19 pandemic ought to be employed more widely in health care. Utilizing these methods could be crucial not only in the post pandemic era and but also under potential public health emergencies.

The REPROGRAM Consortium aimed at analysing and investigating the progress of telemedicine use around the world and the practical challenges the implementation had to face. In another relevant study Baskar el al. confirmed that the increasing evidence gathered suggests a crucial role of telemedicine in improving health system outcomes in many developed countries (76, 77). As a result, international efforts toward developing and using telemedicine during pandemics such as COVID-19, are justified.

More recent research (78) in relation to diabetic care and telemedicine was conducted within the TELEREH-HF (TELEREHabilitation in Heart Failure Patients) trial, a unique comparative initiation employing innovative telemedicine to investigate the efficacy of hybrid comprehensive telerehabilitation (HCTR) on cardiopulmonary exercise capacity in patients with heart failure with reduced ejection fraction (HFrEF) with as opposed to those without diabetes. The effects of a 9-week HCTR were compared to usual care (UC) parameters. The patients were enrolled in the TELEREH-HF trial and randomly placed in the HCTR (N=385 out of which 129 had DM) or usual care (UC) groups (N=397 out of which 137 had DM). Cardiopulmonary exercise tests (CPET) were performed on a treadmill. Among HCTR and UC patients with diabetes, differences in cardiopulmonary test results from baseline to 9 weeks were similar. However, among patients without DM, telerehabilitation generated greater long-term changes in exercise time than usual care. The study concluded that the benefits of HCTR as opposed to UC on the improvement of physical performance, ventilatory profile and gas exchange were more prominent in patients with HFrEF without DM compared to patients with DM. It must be noted, however, that these positive effects did not manifest in clinical outcomes over a follow-up period of 12–24 months in comparison to the usual care groups.

## 8 Tasks and questions for the future

In relation to telemedicine and indeed, in the whole healthcare system, many important questions and issues have arisen that require answers and beg clarification. Some of the most important issues concern availability and access, quality, emerging costs and ethical considerations. Sadly, the access to and use of telemedicine is to a large extent determined by social, economic, and educational factors (79). Extensive research (80) has given account of the importance of prior coaching and training of both patients and medical staff in order for telemedical services to be truly successful. The provision of these will require changes to the mindset of many healthcare professionals and the acquisition of new skill sets. For this reason, we suggest that adequate basic training and continuous education in this field should be a requirement as it is in other areas of medicine even to the extent of needing a licence to practise telemedicine.

Many excellent guidelines have come to light in the recent past about the application and regulation of telemedicine and one prominent study related to diabetology is the professional consensus edited by Zhang (81). This research entails 38 well-defined recommendations along with an evaluation of the evidence concerning the applicability of telemedicine to the whole spectrum of diabetes care from lifestyle therapy to medical treatment. As members of CAPISCO (The CArdiometabolic Panel of International experts on Syndemic COvid-19) (82) we believe that telemedicine is a reliable and useful tool to deliver high-quality patient care. However, we need to consider a number of circumstances. As technology develops, we will be able to monitor even more parameters in real time. Algorithms and artificial intelligence will analyse and interpret the enormous database that is becoming available and as a result, unknown correlations may be revealed in the future. Based on these discoveries, therapy may become even more personalized and, therefore, more effective. At the same time, we must avoid the danger of healthcare becoming illness- and not patient-centric. The first meeting between a doctor and a patient, as well as the initial assessment, should always be conducted in person. If the patient’s condition allows for it and the appropriate technological tools and knowledge are available, telemedicine visits can be added to this in the future. However, in order to assess possible complications, it is still necessary to maintain personal doctor-patient meetings at certain intervals. Many people are afraid that the development of information technology will push personal relationships into the background in healthcare. However, in our opinion, this is just a new communication opportunity, which, if used well, can make doctor-patient meetings more meaningful and, through active involvement of patients, significantly improve the quality of patient care and be more successful in encouraging a healthier lifestyle. Successful treatment has always been based on trust and the relationship between doctors and their patients. It is vitally important that this not be forgotten when discussing and implementing new technologies. This is a key factor that must be borne in mind by both doctor and patient, and that has, up to this point, largely been taken for granted. This may give rise to the most important question for the future: Can this relationship be reformatted? In other words, is it possible to humanize digitalization?

## Author contributions

LR, AM, PK, MR contributed to conception and design of review. All authors contributed to manuscript revision, read, and approved the submitted version.

## References

[1] Zhou F Yu T Du R Fan G Liu Y Liu Z. Clinical course and risk factors for mortality of adult inpatients with COVID-19 in wuhan, China: a retrospective cohort study. Lancet (2020) 395:1054–62. doi: 10.1016/S0140-6736(20)30566-3 PMC7270627 32171076

[2] Chen Y Yang D Cheng B Chen J Peng A Yang C. Clinical characteristics and outcomes of patients with diabetes andCOVID-19 in association with glucose-lowering medication. Diabetes Care (2020) 43:1399–407. doi: 10.2337/dc20-0660 32409498

[3] Mantovani A Byrne CD Zheng M-H Targher G. Diabetes as a risk factor for greater COVID-19 severity and in-hospital death: a meta-analysis of observational studies. Nutr Metab Cardiovasc Dis (2020) 30:1236–48. doi: 10.1016/j.numecd.2020.05.014 PMC7258796 32571616

[4] Holman N Knighton P Kar P O'Keefe J Curley M Weaver A. Risk factors for COVID-19-related mortality in people with type 1 and type 2 diabetes in England: a population-based cohort study. Lancet Diabetes Endocrinol (2020) 8:823–33. doi: 10.1016/S2213-8587(20)30271-0 PMC7426091 32798471

[5] Kempler P Zs Gaál Zs Hermányi Lengyel Cs. COVID-19 és diabétesz: a halálos páros. fókuszban a vércukor. Metabolizmus (2021) 19(2):60–4.

[6] Zhu L She ZG Cheng X Qin JJ Zhang XJ Cai J. Association of blood glucose control and outcomes in patients with COVID-19 and pre-existing type 2 diabetes. Cell Metab (2020) 31:1068–77. doi: 10.1016/j.cmet.2020.04.021 PMC7252168 32369736

[7] WHO. A health telematics policy in support of WHO’s health-For-All strategy for global health development: report of the WHO group consultation on health telematics. Geneva: World Health Organization (1998) p. 11–6.

[8] Vimalananda VG Gupte G Seraj SM Orlander J Berlowitz D Fincke BG. Electronic consulta-tions (e-consults) to improve access to specialty care: a system-atic review and narrative synthesis. J Telemed Telecare (2015) 21:323–30. doi: 10.1177/1357633X15582108 PMC4561452 25995331

[9] Caffery LJ Farjian M Smith AC. Telehealth interventions for reducing waiting lists and waiting times for specialist outpatient services: a scoping review. J Telemed Telecare (2016) 22:504–12. doi: 10.1177/1357633X16670495 27686648

[10] Wade VA Eliott JA Hiller JE. Clinician acceptance is the key factor for sustainable telehealth services. Qual Health Res (2014) 24:682–94. doi: 10.1177/1049732314528809 24685708

[11] Kanat M DeFronzo RA. Abdul-Ghani MA treatment of prediabetes. World J Diabetes (2015) 6(12):1207–22. doi: 10.4239/wjd.v6.i12.1207 PMC4598604 26464759

[12] Tuomilehto J Lindström J Eriksson JG Valle TT Hämäläinen H Ilanne-Parikka P. Prevention of type 2 diabetes mellitus by changes in lifestyle among subjects with impaired glucose tolerance. N Engl J Med (2001) 344:1343–50. doi: 10.1056/NEJM200105033441801 11333990

[13] Lindström J Louheranta A Mannelin M Rastas M Salminen V Tuomilehto J. Lifestyle intervention and 3-year results on diet and physical activitiy. Diabetes Care (2003) 26:3230–36. doi: 10.2337/diacare.26.12.3230 14633807

[14] The Diabetes Prevention Program Research Group. The Diabetes Prevention Program. Design and methods for a clinical trial in the prevention of type 2 diabetes. Diabetes Care (1999) 22(4):623–34. doi: 10.2337/diacare.22.4.623 PMC1351026 10189543

[15] Knowler WC Barrett-Connor E Fowler SE Hamman RF Lachin JM Walker EA. Reduction in theincidence of type 2 diabetes with lifestyle intervention or metformin. N Engl J Med (2002) 346(6):393–403. doi: 10.1056/NEJMoa012512 11832527 PMC1370926

[16] Bian RR Piatt GA Sen A Plegue MA De Michele ML Hafez D. The effect of technology-mediated diabetes prevention interventions on weight: a meta-analysis. J MedInternet Res (2017) 19(3):e76. doi: 10.2196/jmir.4709 PMC5387112 28347972

[17] Grock S Ku JH Kim J Moin T. A review of technology-assisted inter-ventions for diabetes prevention. Curr Diabetes Rep (2017) 17(11):107. doi: 10.1007/s11892-017-0948-2 28942537

[18] Joiner KL Nam S Whittemore R. Lifestyle interventions based on the diabetes prevention program delivered *via* eHealth: a systematic review and meta-analysis. Prev Med (2017) 100:194–207. doi: 10.1016/j.ypmed.2017.04.033 28456513 PMC5699208

[19] Katula JA Dressler EV Kittel CA Harvin LN Almeida FA Wilson KE. Effects of a digital diabetes prevention program: an RCT. Am J Prev Med (2022) 62(4):567–77. doi: 10.1016/j.amepre.2021.10.023 35151522

[20] Almeida FA Michaud TL Wilson KE Schwab RJ Goessl C Porter GC. Preventing diabetes with digital health and coaching for translation and scalability (PRE-DICTS): a type 1 hybrid effectiveness-implementation trial protocol. Contemp Clin Trials (2020) 88:105877. doi: 10.1016/j.cct.2019.105877 31682941

[21] Davis J Fischl AH Beck J Browning L Carter A Condon JE. 2022 National Standards for Diabetes Self-Management Education and Support. Sci Diabetes Self Manag Care (2022) 48(1):44–59. doi: 10.1177/26350106211072203 35049403

[22] Izquierdo RE Knudson PE Meyer S Kearns J Ploutz-Snyder R Weinstock R. A comparison of diabetes education administered through telemedicine versus in person. Diabetes Care (2003) 26:1002–7. doi: 10.2337/diacare.26.4.1002 12663564

[23] Zhang A Wang J Wan X Zhang Z Zhao S Guo Z. A meta-analysis of the effectiveness of telemedicine in glycemic management among patients with type 2 diabetes in primary care. Int J Environ Res Public Health (2022) 19(7):4173. doi: 10.3390/ijerph19074173 35409853 PMC8999008

[24] De Groot J Wu D Flynn D Robertson D Grant G Sun J. Efficacy of telemedicine on glycaemic control in patients with type 2 diabetes: a meta-analysis. World J Diabetes (2021) 12:170–97. doi: 10.4239/wjd.v12.i2.170 PMC7839169 33594336

[25] Little P Stuart B Hobbs FDR Kelly J Smith ER Bradbury KJ. An internet-based intervention with brief nurse support to manage obesity in primary care (POWeR+): a pragmatic, parallel-group, randomised controlled trial. Lancet Diabetes Endocrinol (2016) 4(10):821–28. doi: 10.1016/S2213-8587(16)30099-7 27474214

[26] Pereira K Phillips B Johnson C Vorderstrasse A. Internet Delivered diabetes self-management education: a review. Diabetes Technol Ther (2015) 17(1):55–63. doi: 10.1089/dia.2014.0155 25238257

[27] Ramadas A Quek K Chan C Oldenburg B. Web-based interventions for the management of type 2 diabetes mellitus: asystematic review of recent evidence. Int J Med Inform (2011) 80(6):389–405. doi: 10.1016/j.ijmedinf.2011.02.002 21481632

[28] Poduval S Ross J Pal K Newhouse N Hamilton F Murray E. Web-based structured education for type 2 diabetes: interdisciplinary user-centered design approach. JMIR Hum Factors (2022) 9(1):e31567. doi: 10.2196/31567 35029531 PMC8800092

[29] Dixon RF Zisser H Layne JE Barleen NA Miller DP Moloney DP. A virtual type 2 diabetes clinic using continuous glucose monitoring and endocrinology visits. J Diabetes Sci Technol (2020) 14(5):908–11. doi: 10.1177/1932296819888662 PMC7477772 31762302

[30] Bergenstal RM Layne JE Zisser H Gabbay RA Barleen NA Lee AA. Remote application and use of real-time continuous glucose monitoring by adults with type 2 diabetes in a virtual diabetes clinic. Diabetes Technol Ther (2021) 23(2):128–32. doi: 10.1089/dia.2020.0396 PMC7868574 33026839

[31] Polonsky WH Layne JE Parkin CG Kusiak CM Barleen NA Miller DP. Impact of participation in a virtual diabetes clinic on diabetes-related distress in individuals with type 2 diabetes. Clin Diabetes (2020) 38(4):357–62. doi: 10.2337/cd19-0105 PMC7566922 33132505

[32] Majithia AR Erani DM Kusiak CM Layne JE Lee AA Colangelo FR. Medication optimization among people with type 2 diabetes participating in a continuous glucose monitoring–driven VirtualCare program: prospective study. JMIR Form Res (2022) 6(4):e31629. doi: 10.2196/31629 35147501 PMC9019640

[33] American Diabetes Association. Introduction: standards of medical care in diabetes-2022. Diabetes Care. (2022) 45(Suppl 1):S1–S2. doi: 10.2337/dc22-Sint 34964812

[34] Bollyky JB Bravata D Yang J Williamson M Schneider J. Remote lifestyle coaching plus a connected glucose meter with certified diabetes educator support improves glucose and weight loss for people with type 2 diabetes. J DiabetesRes (2018) 2018:3961730. doi: 10.1155/2018/3961730 PMC5977036 29888288

[35] Garg SK Parkin CG. The emerging role of telemedicine and mobile health technologies in improving diabetes care. Diabetes Technol Ther (2019) 21(S2):S21–3. doi: 10.1089/dia.2019.0090 31169425

[36] Offringa R Sheng T Parks L Clements M Kerr D Greenfield MS. Digital diabetes management application improves glycemic outcomes in people with type 1 and type 2 diabetes. J Diabetes Sci Technol (2018) 12(3):701–8. doi: 10.1177/1932296817747291 PMC6154224 29277103

[37] Khunti S Khunti K Seidu S. Therapeutic inertia in type 2 diabetes: prevalence, causes, consequences and methods to overcome inertia. Ther Adv Endocrinol Metab (2019) 10:2042018819844694. doi: 10.1177/2042018819844694 31105931 PMC6502982

[38] Pantalone KM Misra-Hebert AD Hobbs TM Ji X Kong SX Milinovich A. Antidiabetic treatment patterns and specialty care utilization among patients with type 2 diabetes and cardiovascular disease. Cardiovasc Diabetol (2018) 17(1):54. doi: 10.1186/s12933-018-0699-7 29636104 PMC5892008

[39] Nakamoto S Bitcoin A. A peer-to-Peer electronic cash system. Bitcoin (2008). https://bitcoin.org/bitcoin.pdf, 4(2).

[40] Fang HSA Tan TH Tan YFC Tan CJM. Blockchain personal health records: systematic review. J Med Internet Res (2021) 23(4):e25094. doi: 10.2196/25094 33847591 PMC8080150

[41] Kuo TT Kim HE Ohno-Machado L. Blockchain distributed ledger technologies for biomedical and health care applications. J Am Med Inform Assoc (2017) 24:1211–20. doi: 10.1093/jamia/ocx068 PMC6080687 29016974

[42] Ng WY Tan TE Movva PVH Fang AHS Yeo KK Ho D. Blockchain applications in health care for COVID-19 and beyond: a systematic review. Lancet Digit Health (2021) 3:e819–29. doi: 10.1016/S2589-7500(21)00210-7 PMC8510632 34654686

[43] Chenthara S Ahmed K Wang H Whittaker F Chen Z. Healthchain: a novel framework on privacy preservation of electronic health records using blockchain technology. PloS One (2020) 15:e0243043. doi: 10.1371/journal.pone.0243043 33296379 PMC7725426

[44] Tian H He J Ding Y. Medical data management on blockchain with privacy. J Med Syst (2019) 43:26. doi: 10.1007/s10916-018-1144-x 30603816

[45] Benchoufi M Porcher R Ravaud P. Blockchain protocols in clinical trials: transparency and traceability of consent. F1000 Res (2017) 6:66. doi: 10.12688/f1000research.10531.1 PMC5676196 29167732

[46] Rahman MA Hossain MS Islam MS Alrajeh NA Muhammad G. Secure and provenance enhanced internet of health things framework: a blockchain managed federated learning approach. IEEE Access (2020) 8:205071–87. doi: 10.1109/ACCESS.2020.3037474 PMC8043507 34192116

[47] Polap D Srivastava G Yu K. Agent architecture of an intelligent medical system based on federated learning and blockchain technology. J Inf Secur Appl (2021) 58:102748. doi: 10.1016/j.jisa.2021.102748

[48] Ozercan HI Ileri AM Ayday E Alkan C. Realizing the potential of blockchain technologies in genomics. Genome Res (2018) 28:1255–63. doi: 10.1101/gr.207464.116 PMC6120626 30076130

[49] Aiello M Cavaliere C D’Albore A Salvatore M. The challenges of diagnostic imaging in the era of big data. J Clin Med (2019) 8:316. doi: 10.3390/jcm8030316 30845692 PMC6463157

[50] Ngiam KY Khor IW. Big data and machine learning algorithms for health-care delivery. Lancet Oncol (2019) 20:e262–73. doi: 10.1016/S1470-2045(19)30149-4 31044724

[51] Cichosz SL Stausholm MN Kronborg T Vestergaard P Hejlesen O. How to use blockchain for diabetes health care data and access management: an operational concept. J Diabetes Sci Technol (2019) 13(2):248–53. doi: 10.1177/1932296818790281 PMC6399803 30047789

[52] Borbás J Forczek E Sepp R Bari F. Telecardiology: tasks and du-ties of telemedicine. Orv Hetil (2017) 158(44):1741–6. doi: 10.1556/650.2017.30884 29086593

[53] Bárány T Muk B Osztheimer I Szilágyi Sz Molnár L Kutyifa V. Telecardiology follow-up of pacemaker-implanted patients – inland experiences with home monitoring system. Cardiol Hung (2011) 41:231–8.

[54] Rosta L Mező T Kempler P. Diebetes and COVID-19. demonstration the activity of the Hungarian diabetes association during the pandemic. Diabetologia Hungarica (2022) 30(1):11–8.

[55] The Government of Hungary. National regulation on health ser-vice during the COVID-19 pandemic. Magyar Közlöny (2020) 91:2284–5.

[56] Davis RM Hitch AD Nichols M Rizvi A Salaam M Mayer-Davis EJ. A collaborative approach to the recruitment and retention of minority patients with diabetes in rural community health centers. Contemp Clin Trials (2009) 30(1):63–70. doi: 10.1016/j.cct.2008.09.007 18824135

[57] Shea S Weinstock RS Teresi JA Palmas W Starren J Cimino JJ. Eimicke JPthe IDEATEL consortium a randomized trial comparing telemedicine case management with usual care in older, ethnically diverse, medically underserved patients with diabetes mellitus: 5 year results of the IDEATel study. J Am Med Inform Assoc (2009) 16:446–56. doi: 10.1197/jamia.M3157 PMC2705246 19390093

[58] Davis RM Hitch AD Salaam MM Herman WH Zimmer-Galler IE Mayer-Davis EJ. TeleHealth improves diabetes self-management in an underserved community: diabetes TeleCare. Diabetes Care (2010) 33(8):1712–7. doi: 10.2337/dc09-1919 PMC2909047 20484125

[59] Wosik J Fudim M Cameron B Gellad ZF Cho A Phinney D. Telehealth transformation: COVID-19 and the rise of virtual care. J Am Med Inform Assoc (2020) 27(6):957–62. doi: 10.1093/jamia/ocaa067 PMC7188147 32311034

[60] Patel SY McCoy RG Barnett ML Shah ND Mehrotra A. Diabetes care and glycemic control during the COVID-19 pandemic in the united states. JAMA Intern Med (2021) 181(10):1412–4. doi: 10.1001/jamainternmed.2021.3047 PMC8261690 34228043

[61] Lima-Martínez MM Carrera Boada C Madera-Silva MD Marín W Contreras M. COVID-19 and diabetes: a bidirectional relationship. Clin Investig Arterioscler (2021) 33(3):151–7. doi: 10.1016/j.arteri.2020.10.001 PMC7598432 33303218

[62] Steenblock C Hassanein M Khan EG Yaman M Kamel M Barbir M. Diabetes and COVID-19: short- and long-term consequences. Horm Metab Res (2022) 54(8):503–9. doi: 10.1055/a-1878-9566 PMC9363150 35724689

[63] Boddu SK Aurangabadkar G Kuchay MS. New onset diabetes, type 1 diabetes and COVID-19. Diabetes Metab Syndr (2020) 14(6):2211–7. doi: 10.1016/j.dsx.2020.11.012 PMC7669477 33395782

[64] Jain V Al Rifai M Lee MT Kalra A Petersen LA Vaughan EM. Racial and geographic disparities in Internet use in the U.S. among patients with hypertension or diabetes: implications for telehealth in the era of COVID-19. Diabetes Care (2021) 44(1):e15–7. doi: 10.2337/dc20-2016 PMC7876593 33139408

[65] Garg S Norman GJ. Impact of COVID-19 on health economics and technology of diabetes care: use cases of real-time continuous glucose monitoring to transform health care during a global pandemic. Diabetes Technol Ther (2021) 23(S1):S15–20. doi: 10.1089/dia.2020.0656 PMC7957369 33449822

[66] Khuntia J Ning X Stacey R. Digital orientation of health systems in the post-COVID-19 "New normal" in the united states: cross-sectional survey. J Med Internet Res (2021) 23(8):e30453. doi: 10.2196/30453 34254947 PMC8370259

[67] Kerr D Warshaw H. Clouds and silver linings: COVID-19 pandemic is an opportune moment to democratize diabetes care through telehealth. J Diabetes Sci Technol (2020) 14(6):1107–10. doi: 10.1177/1932296820963630 PMC7645128 33050727

[68] Yildirim P Bozyigit F Ozcanhan MH Utku S. Bulut tabanlı Mobil diyabet kontrol uygulaması: Mobil diyabetim. Bilişim Teknolojileri Dergisi (2017) 10(2):153–59. doi: 10.17671/gazibtd.309295

[69] Ayar D Ozturk M Grey M. The effect of web-based diabetes education on the metabolic control, self-efficacy and quality of life of adolescents with type 1 diabetes mellitus in Turkey. J Pediatr Res (2021) 8(2):131. doi: 10.4274/jpr.galenos.2020.61214

[70] Avdal EU Uran BNO Pamuk G Yildirim JG Konakci G Ates M. Investigation of the effect of web-based diabetes education on metabolic parameters in people with type 2 diabetes: a randomized controlled trial. J Infect Public Health (2020) 13(12):1892–8. doi: 10.1016/j.jiph.2020.03.008 32444190

[71] De Luca V Bozzetto L Giglio C Tramontano G Chiatti C Gonidis F. Satisfaction, self-management and usability: assessment of two novel IT solutions for type 2 diabetes patients’ empowerment. In: Proceedings of the 7th International Conference on Information and Communication Technologies for Ageing Well and e-Health (ICT4AWE 2021) (2021). p. 130–36. doi: 10.5220/0010395901300136

[72] Available at: https://www.resmigazete.gov.tr/eskiler/2022/02/20220210-2.htm.

[73] Available at: https://www.memorial.com.tr/dijital-saglik-asistani.

[74] Aydogan BI Kalemci A Ceylan E Koçyigit D Anil C Demir H. One dose health on-line diabetes follow-up program - preliminary data. In: Proceedings endokurs-6 adana (2022). Abstract 0119 – Adana, Turkey.

[75] Bhaskar S Bradley S Sakhamuri S Moguilner S Chattu VK Pandya S. Designing futuristic telemedicine using artificial intelligence and robotics in the COVID-19 era. Front Public Health (2020) 8:556789. doi: 10.3389/fpubh.2020.556789 33224912 PMC7667043

[76] Bhaskar S Bradley S Chattu VK Adisesh A Nurtazina A Kyrykbayeva S. Telemedicine across the globe-position paper from the COVID-19 pandemic health system resilience PROGRAM (REPROGRAM) international consortium (Part 1). Front Public Health (2020) 8:556720. doi: 10.3389/fpubh.2020.556720 33178656 PMC7596287

[77] Bhaskar S Bradley S Chattu VK Adisesh A Nurtazina A Kyrykbayeva S. Telemedicine as the new outpatient clinic gone digital: position paper from the pandemic health system REsilience PROGRAM (REPROGRAM) international consortium (Part 2). Front Public Health (2020) 8:410. doi: 10.3389/fpubh.2020.00410 33014958 PMC7505101

[78] Główczyńska R Piotrowicz E Szalewska D Piotrowicz R Kowalik I Pencina MJ. Effects of hybrid comprehensive telerehabilitation on cardiopulmonary capacity in heart failure patients depending on diabetes mellitus: subanalysis of the TELEREH-HF randomized clinical trial. Cardiovasc Diabetol (2021) 20(1):106. doi: 10.1186/s12933-021-01292-9 33985509 PMC8120915

[79] Petersen LS Bertelsen P. Equality challenges in the use of eHealth: selected results from a Danish citizens survey. Stud Health Technol Inform (2017) 245:793–7. 29295207

[80] Almathami HK Win KT Vlahu-Gjorgievska E. Barriers and facilitators that influence telemedicine-based, real-time, online consultation at patients’ homes: systematic literature review. J Med Internet Res (2020) 22:e16407. doi: 10.2196/16407 32130131 PMC7059083

[81] Zhang B. Expert consensus on telemedicine management of diabetes (2020 edition). Int J Endocrinol (2021) 2021:6643491. doi: 10.1155/2021/6643491 33833798 PMC8016587

[82] Al Mahmeed W Al-Rasadi K Banerjee Y Ceriello A Cosentino F Galia M. The cardiometabolic panel of international experts on syndemic COVID-19 (CAPISCO). Promoting a syndemic approach for cardiometabolic disease management during COVID-19: the CAPISCO international expert panel front cardiovasc. Med (2021) 8:787761. doi: 10.3389/fcvm.2021.787761 PMC8715947 34977193

